# Supplementary material for: Food and Alcohol Disturbance in High School Adolescents: Prevalence, Characteristics and Association with Problem Drinking and Eating Disorders
Source: Int J Environ Res Public Health. 2024 Jan 11;21(1):83. doi: 10.3390/ijerph21010083 (PMC10815297; doi:10.3390/ijerph21010083)
Supplement: Supplementary file 1 [file ijerph-21-00083-s001.zip › ijerph-2699631-supplementary.pdf]

Supplementary Material

Table S1. Food and Alcohol Disturbance variables definitions in relation to the Compensatory Eating and Behaviors in Response to Alcohol Consumption Scale (CEBRACS).

Variable	Coding	Definition
FAD	0=absent<br>1=present	1=presence of at least 1 item scored ≥ 2<br>0=all items scored = 1
FAD Severity	continuous	total score derived from the sum of the individual items scores (range 21 to 105)
FAD motive	0=absent FAD<br>1= EAE-FAD<br>2= WC-FAD<br>3= EAEWC-FAD)	0=all items scored = 1<br>1= at least 1 item among items 1, 3, 6, 7, 9, 12, 14 scored ≥ 2 and all remaining items scored 1<br>2= at least 1 item among items 2, 4, 5, 8, 10, 11, 13, 15, 16, 17, 18, 19, 20, 21 scored ≥ 2 and all remaining items scored 1<br>3= at least 1 item among items 1, 3, 6, 7, 9, 12, 14 scored ≥ 2 and at least 1 item among items 2, 4, 5, 8, 10, 11, 13, 15, 16, 17, 18, 19, 20, 21 scored ≥ 2
FAD-Before	0=absent<br>1=present	0= items 1 to 6 scored =1<br>1= at least 1 item among items 1 to 6 scored ≥ 2
FAD-During	0=absent<br>1=present	0= items 7 to 14 scored =1<br>1= at least 1 item among items 7 to 14 scored ≥ 2
FAD-After	0=absent<br>1=present	0= items 15 to 21 scored =1<br>1= at least 1 item among items 15 to 21 scored ≥ 2
FAD-Restrictive	0=absent<br>1=present	0= items 4, 10, 11, 16, 20, 21 scored =1<br>1= at least 1 item among items 4, 10, 11, 16, 20, 21 scored ≥ 2
FAD-Hyperactivity	0=absent<br>1=present	0= items 2 and 18 scored =1<br>1= at least 1 item among items 2 and 18 scored ≥ 2
FAD-Purging	0=absent<br>1=present	0= items 5, 8, 13, 15, 17, 19 scored =1<br>1= at least 1 item among items 5, 8, 13, 15, 17, 19 scored ≥ 2

EAE-FAD=FAD behaviors to enhance alcohol effect; WC-FAD=FAD behaviors to control weight; EAEWC-FAD=FAD behaviors to both enhance alcohol effect and control weight; FAD-Before=FAD before alcohol use; FAD-During=FAD during alcohol use; FAD-Ater=FAD after alcohol use; FADrestric tive=restrictng calories; FAD-hyperactivity=physical actvity; FAD-purging=purging behaviors.

Table S2. Results of linear regression analysis with Food and Alcohol Disturbance (FAD, 0=not reported; 1=reported), gender (0=females,1=males), age (continuous) and Lifetime Substance Use (SU, 0=not reported; 1=reported) as predictors and Problem Drinking (PD) Severity as dependent variable. Only participants who reported to engage in FAD behaviors with weight control purposes (WC-FAD and EAEWCFAD) were included. Results are reported for all categories. Significant predictors (α=0.05) are bolded. PD=Problem Drinking; EDR=Eating Disorder Risk.

GROUP	DEPENDENT VARIABLE: PD SEVERITY				
	**Variables**	**B (95% CI); SE**	**β**	**p**	**Adjusted R^2; F; p**
CONTROL (n=638)	(Constant)	-3.542 (-4.564 - -2.521); 0.520		<0.001	0.305; 70.743; <0.001
	**Gender**	0.253 (0.042 - 0.464); 0.107	0.078	0.019	
	**Age**	0.280 (0.217 - 0.343); 0.032	0.293	<0.001	
	**FAD**	1.669 (1.345 - 1.993); 0.165	0.338	<0.001	
	**Lifetime SU**	1.044 (0.716 - 1.372); 0.167	0.211	<0.001	
PD (n=152)	(Constant)	5.705 (-4.996 - 14.093); 5.066		0.262	0.066; 3.646; 0.007
	Gender	1.563 (-0.448 - 2.994); 0.908	0.138	0.087	
	Age	0.125 (-0.354 - 0.763); 0.297	0.034	0.674	
	**FAD**	2.822 (0.939 - 3.998); 0.828	0.271	<0.001	
	Lifetime SU	-0.066 (-1.636 - 1.507); 0.837	-0.006	0.938	
EDR (n=56)	(Constant)	-4.513 (-7.710 - -1.316); 1.592		0.007	0.456; 12.530; <0.001
	Gender	-1.327 (-1.746 – 1.093); 0.707	-0.046	0.646	
	**Age**	0.336 (0.129-0.542); 0.103	0.345	0.002	
	**FAD**	1.748 (0.944 - 2.552); 0.401	0.486	<0.001	
	Lifetime SU	0.312 (-0.776 - 1.400); 0.542	0.061	0.567	

Table S3. Results of linear regression analysis with Food and Alcohol Disturbance (FAD, 0=not reported; 1=reported), gender (0=females,1=males), age (continuous) and Lifetime Substance Use (SU, 0=not reported; 1=reported) as predictors and Eating Disorders (ED) Symptoms Severity as dependent variable. Only participants who reported to engage in FAD behaviors with weight control purposes (WC-FAD and EAEWC-FAD) were included. Results are reported for all categories. Significant predictors (α=0.05) are bolded. PD=Problem Drinking; EDR=Eating Disorder Risk.

GROUP	DEPENDENT VARIABLE: ED SYMPTOMS SEVERITY				
	**Variables**	**B (95% CI); SE**	**β**	**p**	**Adjusted R^2; F; p**
CONTROL (n=638)	(Constant)	36.948 (26.631 – 47.265); 5.254		<0.001	0.193; 39.014; <0.001
	**Gender**	-12.871 (-15.002 - -10.740); 1.085	-0.425	<0.001	
	**Age**	-0.795 (-1.431 - -0.158); 0.324	-0.089	0.015	
	**FAD**	5.815 (2.541 - 9.089); 1.667	0.126	<0.001	
	**Lifetime SU**	3.522 (0.207 - 6.837); 1.688	0.076	0.037	
PD (n=152)	(Constant)	39.595 (15.179 – 64.010); 12.355		0.002	0.315; 18.348; <0.001
	**Gender**	-16.260 (-20.647 - -11.894); 2.214	-0.503	<0.001	
	Age	-0.420 (-1.849 - 1.009); 0.723	-0.040	0.562	
	**FAD**	5.454 (1.462 - 9.447); 2.020	0.184	0.008	
	Lifetime SU	-3.326 (-7.360 - 0.707); 2.041	-0.112	0.105	
EDR (n=56)	(Constant)	72.859 (53.678 - 92.040); 9.554		<0.001	0.011; 1.148; 0.345
	Gender	0.153 (-8.366 – 8.672); 4.243	0.005	0.971	
	Age	-0.117 (-1.357 - 1.123); 0.618	-0.027	0.850	
	FAD	-4.537 (-9.362 - 0.288); 2.403	-0.284	0.065	
	Lifetime SU	0.538 (-5.991 – 7.067); 3.252	0.023	0.869	
